# Optimal harvesting of a continuously age-structured population with density dependence

**DOI:** 10.1371/journal.pone.0333087

**Published:** 2025-09-25

**Authors:** Mark Opmeer

**Affiliations:** Department of Mathematical Sciences, University of Bath, Bath, United Kingdom; Wildlife Conservation Society Canada, CANADA

## Abstract

We consider harvesting of a population with continuous age-structure and where density dependence is implemented through interaction of the population with a food source. Using a Von Bertalanffy length-age relation, the continuous age-structure is equivalent to a continuous length-structure. We allow the harvesting rate to be an arbitrary function of length. This allows for a comparison of harvesting strategies, including conventional harvesting and balanced harvesting. As a particular example, we consider plaice (*Pleuronectes platessa*, Pleuronectidae). The harvesting rate which gives the maximum sustainable yield is consistent with conventional harvesting: there exists a body size such that individuals smaller than that size are not harvested and individuals larger than that size are maximally harvested.

## Introduction

In the conventional theory of fishing [1], it is assumed that there exists a length (*length at first capture*) $\ell_{c}$ below which the fishing mortality rate equals zero and above which the fishing mortality rate equals some constant *F*_0_. An objective in the conventional theory of fishing then is to determine the pair $\left(\ell_{c},F_{0}\right)$ which achieves Maximum Sustainable Yield (MSY).

There has recently been a proposal for “balanced harvesting” as an alternative to this conventional harvesting [2,3]. There is some confusion about what balanced harvesting precisely means [4, page 212]. It is alternatively presented as a harvesting policy which preserves ecosystem structure or as a harvesting policy where fishing mortality rate is proportional to productivity. Moreover, “ecosystem structure” and “productivity” themselves are multi-interpretable. Several different possible implementations of the idea are considered in [5]. See [6] for a critique of balanced harvesting. It is however clear that balanced harvesting is considered as different from conventional harvesting and therefore that it proposes a fishing mortality rate which is not of the conventional form described above.

**Fig 1:**
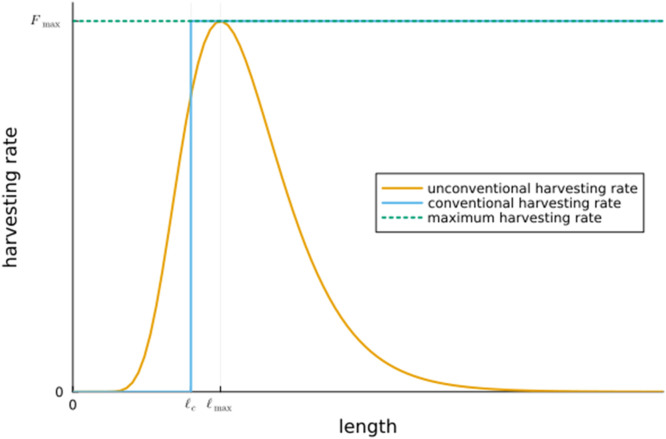
For a given maximal harvesting rate $F_{\max}$, conventional harvesting gives a zero harvesting rate below length $\ell_{c}$ and a harvesting rate $F_{\max}$ above $\ell_{c}$. Also depicted is a harvesting rate which is not of the conventional type as a function of length as $F(\ell )=F_{\text{max}}\exp(-\frac{1}{2\sigma_{m}}{\left(\ln(\frac{\ell^{3}}{\ell_{\text{max}}^{3}})\right)}^{2})$ for some parameters $\sigma_{m}$ and $\ell_{\max}$.

We consider a harvesting rate *F* which can be an arbitrary function of length ℓ satisfying $0\leq F(\ell )\leq F_{\max}$ for some maximal rate $F_{\max}>0$. This in particular includes both conventional harvesting and balanced harvesting (however implemented) and other harvesting strategies as depicted in Fig 1 as possibilities. We emphasize that in our setting, finding the harvesting rate which achieves Maximum Sustainable Yield is not a parameter optimization problem, but instead the optimization is over all functions *F* satisfying $0\leq F(\ell )\leq F_{\max}$. We show analytically that the harvesting rate which achieves MSY at each length equals either 0 or $F_{\max}$. This in particular implies that unconventional harvesting as depicted in Fig 1 does not achieve MSY. For parameter values corresponding to plaice (*Pleuronectes platessa*, Pleuronectidae) we show numerically that the MSY harvesting rate is of the conventional form: there exists a length $\ell_{c}$ such that F(ℓ) equals zero for $\ell <\ell_{c}$ and equals $F_{\max}$ for $\ell >\ell_{c}$.

We emphasize that in contrast to the conventional theory of fishing [1], we do not *assume* that the harvesting rate is of this form, it is instead a *consequence* of the wish to maximize sustainable yield.

## Methods

### Model description

Let *n*(*t*,*a*) be the density of a population at time *t* with age *a* so that


$$\int_{a_{1}}^{a_{2}}n(t,a)\;da,$$

is the size of the population at time *t* with ages in between *a*_1_ and *a*_2_. The classical McKendrick–Von Foerster equation describing the time evolution of *n* is


$$\frac{\partial n}{\partial t}+\frac{\partial n}{\partial a}=-Z(t,a)n,$$

where *Z*(*t*,*a*) > 0 is the mortality rate. We have


Z(t,a)=M+F(t,a),

where *M* > 0 is the natural mortality rate (assumed to be constant) and F(t,a)≥0 is the harvesting rate. Harvesting is usually body-size-dependent rather than age-dependent. Therefore we consider an age-length relationship as follows:


$$\frac{\partial \ell }{\partial t}+\frac{\partial \ell }{\partial a}=g(\ell ),\;\ell (t,a=0)=\ell_{b},$$

where ℓ(t,a) denotes the length at time *t* and age *a*, $\ell_{b}>0$ is the length at birth (“birth” in our context of plaice actually means benthic settlement), and *g* is a given growth function. Since *g* and $\ell_{b}$ are assumed to be independent of time *t*, it follows that ℓ is in fact independent of *t* as well. In the special case that $g(\ell )=K(\ell_{\infty }-\ell )$, where $K,\ell_{\infty }>0$ we have that

$$\ell (a)=\ell_{\infty }-(\ell_{\infty }-\ell_{b}){\text{e}}^{-Ka},$$ (1)

which is the classical Von Bertalanffy relation. From this we obtain age as a function of length


$$a(\ell )=\frac{1}{K}\ln\left(\frac{\ell_{\infty }-\ell_{b}}{\ell_{\infty }-\ell }\right).$$

The applicability of the Von Bertalanffy relation is extensively discussed in [1].

We assume that a reproduction function β(a) is given with the interpretation that


$$\int_{a_{1}}^{a_{2}}\beta (a)n(t,a)\;da,$$

is the total number of births at time *t* to parents with age in between *a*_1_ and *a*_2_. Reproduction is in fact better described in terms of length than in terms of age, so we will prescribe the reproduction function *β* as a function of length and β(a) in the above is in fact β(ℓ(a)). As is common, we assume that there is a length at maturation $\ell_{m}>0$ below which no reproduction takes place. We take


$$\beta (\ell )=\left\{ \begin{array}{cc} 0 & \ell <\ell_{m}\\ r\ell^{3} & \ell \geq \ell_{m}, \end{array}\right.$$

where *r* > 0 is a fecundity coefficient. That reproduction is proportional to $\ell^{3}$ (and hence to volume and weight) is a common assumption [1, Section 6.1.2] which is reasonably supported by data [7].

It follows from integrating the McKendrick–Von Foerster equation over all ages that


$$\frac{d}{dt}\int_{0}^{\infty }n(t,a)\;da=-\int_{0}^{\infty }\frac{\partial n}{\partial a}\;da-\int_{0}^{\infty }Z(t,a)n(t,a)\;da.$$

Since the left-hand side is the change in time of the total population and the second term on the right equals total mortality, the first term on the right must equal total births (assuming a closed system, i.e. with no migration). It follows that we must have


$$\int_{0}^{\infty }\beta (a)n(t,a)\;da=-\int_{0}^{\infty }\frac{\partial n}{\partial a}\;da,$$

i.e.


$$\int_{0}^{\infty }\beta (a)n(t,a)+\frac{\partial n}{\partial a}\;da=0.$$

### Incorporating density dependence

In accordance with [8,9] we assume that the growth function *g* and the reproduction function *β* depend on a variable *z* (which can be given the interpretation of a food source). This will result in density-dependence. More precisely we assume that


$$\ell_{\infty }=\ell_{\max}f(z),\;r=r_{0}f(z),$$

i.e. that


$$g(\ell ,z)=K\left(\ell_{\max}f(z)-\ell \right),\;\beta (\ell ,z)=\left\{ \begin{array}{cc} 0 & \ell <\ell_{m}\\ r_{0}f(z)\ell^{3} & \ell \geq \ell_{m}, \end{array}\right.$$

where $\ell_{\max}>0$, *r*_0_>0, and *f* has the Beverton–Holt form $f(z)=\frac{z}{z+z_{h}}$, where *z*_*h*_>0 is given. The variable *z* is assumed to satisfy


$$\frac{dz}{dt}=h(z)-I(t),$$

where *I*(*t*) is the (density-dependent) consumption at time *t* and is given by


$$I(t)=\int_{0}^{\infty }I_{\max}f(z)n(t,a)\;\ell (a{)}^{2}\;da,$$

where $I_{\max}>0$ and *h* determines the time evolution of *z* when *n* = 0 for which we will assume the chemostat form h(z)=ν(C−z) where ν,C>0. That consumption is proportional to $\ell^{2}$ (and hence to area) is a common assumption which is reasonably supported by data [1, Section 9.4.3.1.1].

That $\ell_{\infty }$ depends on food consumption, but *K* does not is consistent with [1, Section 9.4.1].

The total yield *Y*(*t*) at time *t* from harvesting is


$$Y(t)=\int_{0}^{\infty }\delta_{v}F(t,a)n(t,a){\left(\delta_{m}\ell (a)\right)}^{3}\;da,$$

where $\delta_{v}>0$ is volume specific mass and $\delta_{m}>0$ is a shape coefficient.

We are interested in maximum sustainable yield, so we consider the situation where everything is independent of time *t* and we wish to maximize the yield in this situation. For numerical considerations we replace the upper-limit ∞ in the integrals in the above description by some large finite $a_{\max}$ where $a_{\max}>0$ is a maximal age. We further assume that the harvesting rate is bounded by some $F_{\max}>0$, i.e. $0\leq F(a)\leq F_{\max}$. We will comment on the role of $F_{\max}$ in the results section.

We summarize the problem in (2).



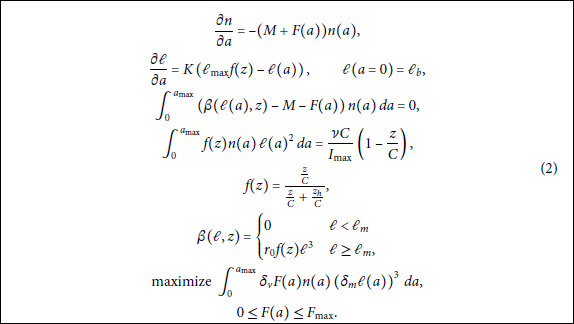



### Parametrization

We consider parameter values for plaice (*Pleuronectes platessa*, Pleuronectidae), but other species could similarly be considered. From FishBase [10] we obtain the following


$$\ell_{\max}=100\text{\textasciitilde{}cm},\;M=0.12{\text{\textasciitilde{}year}}^{-1},\;K=0.1{\text{\textasciitilde{}year}}^{-1},\;\ell_{m}=26.6\text{\textasciitilde{}cm}.$$

We further assume (in accordance with [9])


$$\ell_{b}=1\text{\textasciitilde{}cm},\;a_{\max}=30\text{\textasciitilde{}year}.$$

We have that $\frac{\nu C}{I_{\max}}$ only scales the density *n*, so that we can assume without loss of generality that it equals 1 (by considering *n* in appropriate units). Similarly, $\delta_{v}$ and $\delta_{m}$ scale out of the problem.

The lifetime reproductive output in the density-independent case (*f* = 1) and with no harvesting (*F* = 0) equals


$$r_{0}\int_{a_{m}}^{a_{\max}}{\text{e}}^{-Ma}{\left(\ell_{\max}-(\ell_{\max}-\ell_{b}){\text{e}}^{-Ka}\right)}^{3}\;da,$$

where *a*_*m*_ is the age at maturity in this context, i.e.


$$a_{m}=\frac{1}{K}\ln\left(\frac{\ell_{\max}-\ell_{b}}{\ell_{\max}-\ell_{m}}\right).$$

We choose *r*_0_ so that this lifetime reproductive output equals 22.2 [11]. This gives


$$r_{0}=1.49\times 10^{-5}.$$

The parameter $z_{h0}:=\frac{z_{h}}{C}$ is chosen as 0.25.

### Formulation as a standard optimal control problem

We can reformulate the maximum sustainable yield problem (2) as a standard optimal control problem. The independent variable is age *a*, the control variable *u* is the harvesting rate *F* and we define the state *x* (which is a vector with 4 components) through


$$x_{1}:=n,\;x_{2}:=\ell ,\;x_{3}(A):=\int_{0}^{A}\left(\beta (\ell (a),z_{0})-M-F(a)\right)n(a)\;da,x_{4}(A):=\int_{0}^{A}f(z_{0})n(a)\;\ell (a{)}^{2}\;da.$$

With these definitions, (2) becomes the following standard optimal control problem:



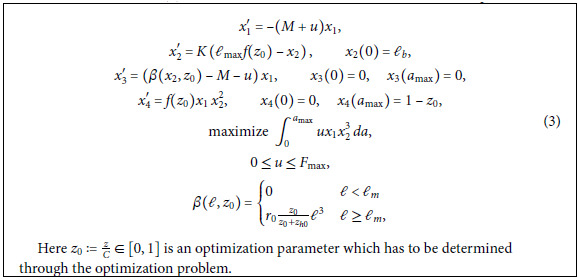



### Analysis of the optimal control problem

The Pontryagin Principle [12–14] can be used to obtain information about the optimal control problem (3). The Hamiltonian of the problem equals (for notational convenience, we suppress dependence on *z*_0_)


$$-ux_{1}x_{2}^{3}-\lambda_{1}(M+u)x_{1}-\lambda_{2}K\left(x_{2}-f\ell_{\max}\right)-\lambda_{3}\left(M+u-\beta (x_{2})\right)x_{1}+\lambda_{4}I_{\max}fx_{1}x_{2}^{2}.$$

Defining the switching function (the coefficient of *u* in the Hamiltonian)


$$\sigma :=-(x_{2}^{3}+\lambda_{1}+\lambda_{3})x_{1},$$

we have that


$$u(a)=\left\{ \begin{array}{cc} 0 & \sigma (a)>0\\ \text{singular} & \sigma (a)=0\\ F_{\max} & \sigma (a)<0. \end{array}\right.$$

We further have


$$\lambda_{1}^{\prime }=ux_{2}^{3}+\lambda_{1}(M+u)+\lambda_{3}\left(M+u-\beta (x_{2})\right)-\lambda_{4}I_{\max}fx_{2}^{2},\;\lambda_{1}(0)=\lambda_{1}(a_{\max})=0,\;\lambda_{3}^{\prime }=0,\;\lambda_{4}^{\prime }=0.$$

From this we can deduce that


$$\sigma^{\prime }=\left(Mx_{2}^{3}-3x_{2}^{2}K\left(f\ell_{\max}-x_{2}\right)+\lambda_{3}\beta (x_{2})+\lambda_{4}I_{\max}fx_{2}^{2}\right)x_{1}.$$

We now exclude the case of singular control. In the case of singular control we have σ=0 on a non-trivial interval and therefore $\sigma^{\prime }=0$ on that interval. Since *x*_1_>0, it follows that on that interval


$$Mx_{2}^{3}-3x_{2}^{2}K\left(f\ell_{\max}-x_{2}\right)+\lambda_{3}\beta (x_{2})+\lambda_{4}I_{\max}fx_{2}^{2}=0.$$

Since $\lambda_{3}$ and $\lambda_{4}$ are constants, it follows from this that *x*_2_ must be piecewise constant on this interval. However, since *x*_2_ is given by the Von Bertalanffy relation (1), this is not possible. It follows that singular control is impossible and that therefore we have bang-bang control, i.e.


$$u(a)=\left\{ \begin{array}{cc} 0 & \sigma (a)>0\\ F_{\max} & \sigma (a)<0. \end{array}\right.$$

Hence for any given age (or equivalently: length) the harvesting rate is either zero or maximal.

### Numerical considerations

For numerical purposes it is needed to approximate the discontinuous reproduction function *β* by a smooth one. The numerical method therefore uses instead


$$\beta (\ell ,z)=r_{0}f(z)\ell^{3}\frac{1+\frac{1}{2}{\text{e}}^{-10(\ell -\ell_{m})}}{1+{\text{e}}^{-10(\ell -\ell_{m})}},$$

utilizing a standard smooth approximation of the sign function. Note that the argumentation in the previous section excluding singular control carries over since this didn’t use any specific form for *β*.

The numerical solution of (3) starts from an initial guess for the control, the state and the optimization parameter. We use


$$u_{\text{init}}(a)=\frac{F_{\max}}{2},\;z_{0,\text{init}}=z_{h0},\;x_{1,\text{init}}(a)={\text{e}}^{-(M+F_{\max}/2)a},$$

$$x_{3,\text{init}}(a)=\frac{\ell_{\max}}{2}-\left(\frac{\ell_{\max}}{2}-\ell_{b}\right){\text{e}}^{-Ka},\;x_{4,\text{init}}=0,\;x_{4,\text{init}s}=1-z_{h0}.$$

A very bad initial guess will lead to non-convergence or convergence to the zero solution. However, a large range of initial guesses leads to the solution mentioned in the results section (and no solution other than this solution or the zero solution was ever found).

Note that the above initial guess does not bias towards *u* having the conventional harvesting form.

## Results

**Fig 2:**
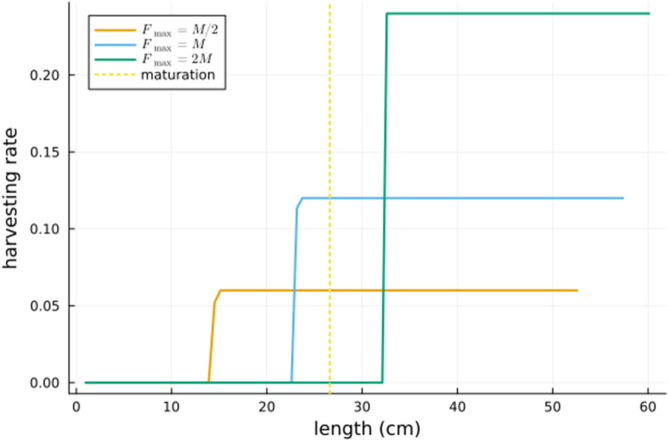
The optimal harvesting rate as a function of length for three values of the maximum harvesting rate $F_{\max}$. Also shown is the length of maturation.

The numerical calculations were performed with the Julia package OptimalControl.jl [15]. For values of the maximum harvesting rate $F_{\max}$ in $\left\{\frac{M}{2},M,2M\right\}$ the optimal harvesting rate as a function of length is depicted in Fig 2. It can be seen that the optimal harvesting rate is zero below a certain length $\ell_{c}$ and is maximal above this length. This switching length $\ell_{c}$ depends on $F_{\max}$ and increases with $F_{\max}$.

**Fig 3:**
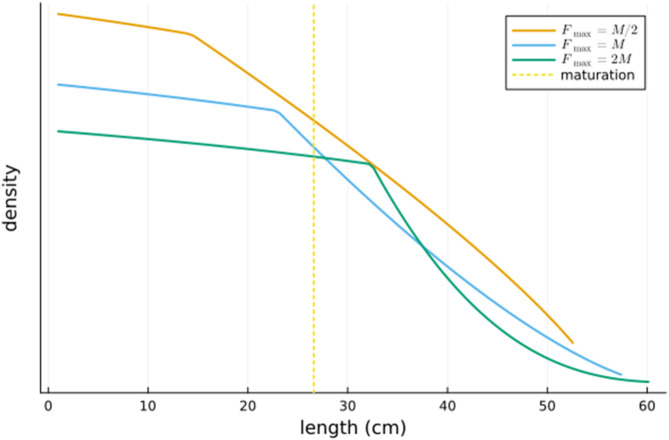
The optimal density as a function of length for three values of the maximum harvesting rate $F_{\max}$. Also shown is the length of maturation.

By the chain rule we have


$$\int_{a_{1}}^{a_{2}}n(a)\;da=\int_{\ell (a_{1})}^{\ell (a_{2})}n(a(\ell ))\;\frac{da}{d\ell }\;d\ell =\int_{\ell (a_{1})}^{\ell (a_{2})}n(a(\ell ))\;\frac{1}{g(\ell )}\;d\ell ,$$

so that $N(\ell ):=n(a(\ell ))\;\frac{1}{g(\ell )}$ is the density as a function of ℓ in the sense that


$$\int_{\ell_{1}}^{\ell_{2}}N(\ell )\;d\ell ,$$

is the size of the population with lengths in between $\ell_{1}$ and $\ell_{2}$. In Fig 3 we give this density as a function of length for the above three values of the maximum harvesting rate $F_{\max}$.

**Fig 4:**
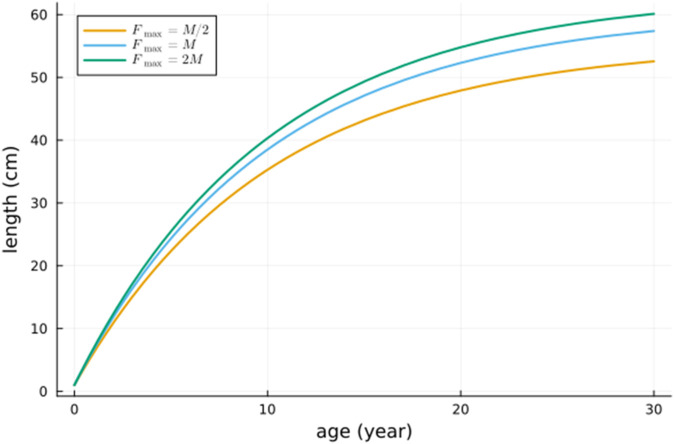
Length as a function of age for three values of the maximum harvesting rate $F_{\max}$.

Length as a function of age is depicted in Fig 4. We note that because of density dependence the ultimate length $\ell_{\infty }$ depends on the variable *z*. What we see in Fig 4 is that the ultimate length increases with the maximal harvesting rate $F_{\max}$. This is because the variable *z* increases with $F_{\max}$ and $\ell_{\infty }$ increases with *z*.

### The role of the maximum harvesting rate

It is not our main focus, but one might wonder about how the maximum harvesting rate $F_{\max}$ (which could be seen as being proportional to the size of the fishing fleet) influences the results.

Because of the nature of the problem, the maximum sustainable yield is increasing with the maximum harvesting rate $F_{\max}$. As indicated in Table 1, beyond a value of $F_{\max}=5M$ there is hardly any improvement in the maximum sustainable yield.

**Table 1:** The maximum sustainable yield for various values of the maximum harvesting rate $F_{\max}$ normalized by the maximum sustainable yield when the maximum harvesting rate is equal to the natural mortality rate *M.*

$F_{\max}/M$	MSY($F_{\max}$ )/MSY(*M*)
$\frac{1}{2}$	0.662
1	1
2	1.219
5	1.315
10	1.333
20	1.338
30	1.339

**Fig 5:**
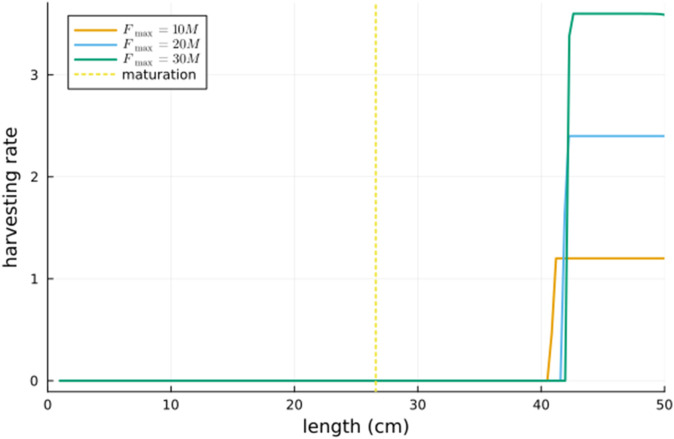
The optimal harvesting rate as a function of length for three large values of the maximum harvesting rate $F_{\max}$. Also shown is the length of maturation.

**Fig 6:**
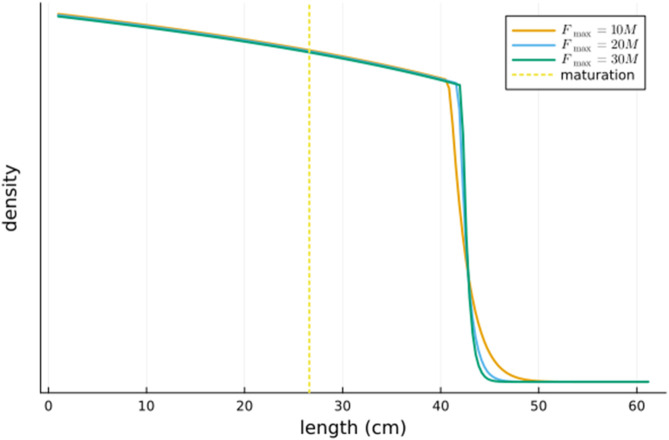
The optimal density as a function of length for three large values of the maximum harvesting rate $F_{\max}$. Also shown is the length of maturation.

For relatively large values of the fraction $\frac{F_{\max}}{M}$, we depict the optimal harvesting rate as a function of length in Fig 5 and the density as a function of length in Fig 6. It seems that as $\frac{F_{\max}}{M}\to \infty$, the switching length $\ell_{c}$ converges to some finite value $\ell_{c}^{*}$ (this is consistent with [1, Figure 17.14] which considers a somewhat different model) and that the optimal harvesting strategy is to harvest the whole population with length above $\ell_{c}^{*}$ (and harvest none with length below this).

## Discussion

We considered harvesting of a population with length as a continuous variable and density-dependence through interaction with a food source. We allowed the harvesting rate to be an arbitrary function of length. The optimal harvesting rate (with respect to MSY) turned out to be of the form used in conventional harvesting of fish: individuals below a certain body size are not harvested and those above that size are (maximally) harvested. This is an argument in favor of conventional harvesting and against balanced harvesting (we note that balanced harvesting, however implemented, is mathematically guaranteed not to give maximum sustainable yield for our model). We do consider a single species model (interacting with a food source) and the results could potentially be different if a model of several interacting species were to be considered instead. The optimal control problem could be generalized to include such interaction. Optimal harvesting problems are linear in the control and therefore belong to a very special class of optimal control problems. The only way that an optimal harvesting rate which does not equal either zero or maximal for all lengths can arise in such a problem is as singular control. For our model this has been mathematically excluded, but it is in principle possible that singular control is optimal for other models (for example multi-species models). This is the mathematical mechanism through which something that might be termed balanced harvesting could be optimal: as singular control.

Instead of maximum sustainable yield as objective, one could consider maximum economic sustainable yield by adding $-cF_{\max}$ to the objective function for some *c* > 0 (this includes cost of harvesting in the objective). The maximal harvesting rate $F_{\max}$ should then be treated as an optimization parameter (similarly to the variable *z* in (2) or the normalized variable *z*_0_ in (3)). Due to the diminishing returns in yield with increasing $F_{\max}$ shown in Table 1, there will exist a unique $F_{\max}^{*}$ which gives the maximum economic sustainable yield. The optimal harvesting rate and optimal density will then be as calculated in the results section with the maximal harvesting rate set equal to this $F_{\max}^{*}$. Therefore in essence, we have also solved the maximum economic sustainable yield problem.

We provided a partial mathematical analysis of the optimal control problem (3). Fuller mathematical analysis of similar problems is available in the literature [16], but is seems not of our exact problem. This mathematical analysis of similar, but different, problems is consistent with our results in that it excludes singular control and typically finds that there exists one switch between minimal and maximal control.

It is easy to incorporate additional constraints into the optimal control problem, for example those proposed in [17].

## Supporting information

S1 File Julia-Fish.jl The Julia code.
(JL)
